# Assessing the Impact of Color Normalization in Convolutional Neural Network-Based Nuclei Segmentation Frameworks

**DOI:** 10.3389/fbioe.2019.00300

**Published:** 2019-11-01

**Authors:** Justin Tyler Pontalba, Thomas Gwynne-Timothy, Ephraim David, Kiran Jakate, Dimitrios Androutsos, April Khademi

**Affiliations:** ^1^Image Analysis in Medicine Lab (IAMLAB), Ryerson University, Toronto, ON, Canada; ^2^Pathcore Inc., Toronto, ON, Canada; ^3^St. Michael's Hospital, Toronto, ON, Canada

**Keywords:** computational pathology, standardization, neural networks, deep learning, color normalization, nuclei segmentation

## Abstract

Image analysis tools for cancer, such as automatic nuclei segmentation, are impacted by the inherent variation contained in pathology image data. Convolutional neural networks (CNN), demonstrate success in generalizing to variable data, illustrating great potential as a solution to the problem of data variability. In some CNN-based segmentation works for digital pathology, authors apply color normalization (CN) to reduce color variability of data as a preprocessing step prior to prediction, while others do not. Both approaches achieve reasonable performance and yet, the reasoning for utilizing this step has not been justified. It is therefore important to evaluate the necessity and impact of CN for deep learning frameworks, and its effect on downstream processes. In this paper, we evaluate the effect of popular CN methods on CNN-based nuclei segmentation frameworks.

## Introduction

In 2015, the World Health Organization (WHO) estimated that cancer was the leading cause of death in 91 of 172 countries. By the end of 2018, there was an estimated 18.1 million new cancer cases, and 9.6 million cancer related deaths (Bray et al., 2018). Cancer is prevalent worldwide, and while the causes are not yet fully known, several risk factors have been identified through routine analysis of clinical data (Maringe et al., 2013).

An important tool for the detection and management of cancer is the analysis of tissue samples under assessment by a pathologist (Hutter, 1991). Based on visual analysis of the tissue and cells, the pathologist renders a diagnosis, determines the aggressiveness of the disease, and recommends a treatment plan. Pathological analysis of tissue slides, in the form of histological grading, is critical to cancer treatment planning and for delivering high quality patient care.

Histological grading is an important practice that describes how abnormal tumor cells and tumor tissue appear under a microscope. It is common among all cancers and involves the analysis of tissue specimens for characteristics of malignancy. In preparation for grading, hematoxylin, and eosin (H&E) dyes are used to increase tissue contrast by highlighting specific structures. For instance, hematoxylin, normally purple, is a stain that has an affinity to the nucleic acids contained in the nuclei. Eosin, normally pink, is a counter stain that binds to the cytoplasm of cells (Hortobagyi et al., 2017). The combination of the two stains improves contrast and makes it easier to discern cell and tissue characteristics. Characteristics such as cell appearance, nuclear pleomorphism (size and shape), and spatial arrangement of cells are important metrics for determining the histological grade (Hortobagyi et al., 2017). However, conventional grading is time consuming, and the interpretation is subjective and error-prone (Rakha et al., 2010; Khademi, 2013).

Nuclear grading is common between many cancer grading systems, and examines the appearance and morphology of cells. Unfortunately, grading for nuclear pleomorphism suffers greatly from interpretation variability. For example, using the Nottingham Grading System for breast cancer, nuclear grading has poor-to-moderate agreement (Andrion et al., 1995). Similar results are found for other cancers as described in Wludarski et al. (2011) and Ismail et al. (1989). As such, automating pathological analysis for nuclear grading proposes the opportunity to reduce subjectivity, variability, and workload, and in turn, increase reliability, reproducibility, and improve clinical workflow.

The FDA approved the first, clinical, whole-slide imaging (WSI) scanner in April 2017, which was a milestone for transitioning anatomical pathology to a digital practice (Boyce, 2017). Digital images produced by WSI scanners enable clinicians to visualize cellular and tissue microstructure in full color and under high resolution. In addition, digital image data enables the use of automated image analysis and machine learning tools, broadly computational pathology, to improve the accuracy and efficiency of nuclear grading systems. Early automated tools included nuclei detection, but more recently nuclei segmentation has become more important, since features from entire nuclei can be extracted and analyzed.

Automated segmentation of nuclei is one of the most crucial steps for automated nuclear grading systems and has remained challenging due to the complexity of the task. Firstly, the characteristics of the cells are quite variable from patient to patient. Cancerous nuclei can be highly pleomorphic and tumors often display heterogeneity. Reviews state that traditional segmentation frameworks have poor segmentation accuracy for images with cancerous nuclei (Cloppet and Boucher, 2009; Di Cataldo et al., 2010). This is especially apparent when the nuclei are clustered and overlapping (Wählby et al., 2004; Cloppet and Boucher, 2009; Di Cataldo et al., 2010). Some of the traditional nuclei segmentation methods include morphological processing (Loménie and Racoceanu, 2012), hand-crafted feature design and classification (Hasan and Roy-chowdhury, 2014), unsupervised clustering (Parvin et al., 2007), and supervised approaches that classify each pixel into different categories: nuclei or background (Mouelhi et al., 2013; Xu et al., 2016a; Bejnordi et al., 2017). Throughout these frameworks, data variability and algorithm generalization continue to be the main barrier.

Over the last few years, deep learning (DL)-based algorithms, such as convolutional neural networks (CNN), have become popular in the analysis of digital tissue specimens. DL frameworks demonstrate dominating performance in generalizing to highly variable data (Al-Milaji et al., 2017). This makes them suitable for computational pathology applications such as segmentation. DL-based methods for region-specific or object-level segmentation have been proposed in several works (Shelhamer et al., 2014; Ronneberger et al., 2015; Xu et al., 2015, 2016b; Chen et al., 2016; Agarwalla et al., 2017; Al-Milaji et al., 2017; Kumar et al., 2017; Li et al., 2017; Naylor et al., 2017; Alom et al., 2018; De Xie, 2018; Graham and Rajpoot, 2018; Wang et al., 2019).

An early DL framework, proposed by Ronneberger et al. segmented cells in electron microscopy images by using a variation of a fully convolutional neural network (FCN)—also known as “U-Net.” The original FCN architecture utilizes a series of successive convolution layers and max pooling operations, followed by subsequent up-sampling and additional convolution layers. U-Net was developed by connecting features from the downward path with up-sampled outputs at various layers. By connecting these paths, high resolution features can be localized at the output layers. The work contributed by Ronneberger et al. has been adapted and improved for semantic region and object-level segmentation for H&E stained images. For instance, Li et al. altered the original U-Net architecture by introducing multi-scale image patches into the training set. Incorporating the same region at three different sizes provides contextual information to the network and resulted in a greater gland segmentation accuracy compared to the original U-Net (Li et al., 2017). In addition, Alom et al. improved segmentation results by replacing the forward convolutional layers with recurrent convolutional layers. This replacement, along with accumulating features outside the network, and replacing “cropping and copying” operations with concatenation, improved nuclei segmentation accuracy (Alom et al., 2018).

Classic deep learning frameworks with some modifications to parameters, hyper-parameters, and post-processing techniques, continue to be implemented and demonstrate success for region specific and pixel-wise segmentation in digital H&E images. Xu et al., Kumar et al., Agarwalla et al., Al-Milaji et al., and Xie et al., implement various CNN architectures that perform one, or both, region specific and pixel-wise segmentation. As demonstrated by the number of different architectures that exist in literature, there are numerous DL-based methods that address the issue of *data variability* in segmentation tasks and attempt to improve generalizability for multicenter data.

In multicenter digital pathology datasets, there is the problem of *color constancy*, which is attributed to the lack of standardization in laboratory staining practices, and the inherent variation contributed by the multitude of dye and digital scanner manufacturers (Macenko et al., 2009). In such samples, the color characteristics of cells and tissue can vary drastically across imaging centers, even for the same tissue types and stains. As computational algorithms begin to expand to the clinical domain, algorithms that generalize and scale to large, multi-institutional datasets are needed to fully realize the potential of AI in digital pathology.

For classic segmentation methods, instead of developing many models that handle the different degrees of variability, *color normalization (CN)* is applied as a preprocessing step. CN is common preprocessing technique that attempts to reduce color variability and improve the generalization of algorithms by transforming the input data to a common space. In color normalized digital pathology samples, regions of digital tissue specimens are mapped to similar color characteristics regardless of the scanning device, stain vendor, and preparation protocols. Because of the reduced variability in color characteristics of tissues, CN has demonstrated improvement in computer-assisted diagnostic tools (CADs) (Khan et al., 2014; Bejnordi et al., 2016; Kumar et al., 2017; Li et al., 2018).

While CN is often used in non-DL segmentation frameworks, there are DL architectures that utilize CN as a pre-processing technique as well (Kumar et al., 2017; Li et al., 2018). However, when CN has been applied in DL-based frameworks, the necessity and effect of CN has traditionally not been evaluated. In order for DL-based frameworks to generalize to multicenter data effectively, the effect of color variability and subsequently, CN as a preprocessing step, needs investigation. To our knowledge there are no works to date that systematically assess the effect of CN on DL-based frameworks—and is the subject of this work.

In this work, we extend the DL-based nuclei segmentation methods of Kumar et al. by implementing a *ternary* segmentation scheme (nuclei, boundary, background) on two different CNN architectures to assess the effect of CN on model performance. In particular, a patch-based CNN and the UNET architecture are evaluated, and five open source CN methods are used to normalize the training and test datasets. A reference image is used for CN in all methods except for the generative adversarial network (GAN)-based method, which uses a collection of images. The effectiveness of each CN scheme is evaluated using image quality metrics, including a novel metric proposed called the normalized median hue (NMH), which quantifies the global color variation of an image population.

Using models generated from the un-normalized and CN data, the segmentation results from all CN methods are compared to results generated by the un-normalized model using various overlap metrics. An ensemble nuclei segmentation model is also proposed, that combines the results from the various CN models per architecture to investigate whether these classifiers combined can improve segmentation performance. To address the multicenter data problem, we evaluate the performance of the nuclei segmentation models on various H&E datasets, each with unique color characteristics. In total, three datasets are used. The first one is “TCGA-Kumar” and it is tissue of the seven different types and there are 29 images. These images contain ternary annotations and are used for training (with 7 held out for testing). The second one, “TNBC” contains 50 images of triple negative breast cancer tissue. The last dataset, “SMH,” is contains images of lymph node tissue from patients with suspected breast cancer metastases. These datasets comprise variable color characteristics and are ideal for testing the generalizability and clinical utility of the suggested framework.

The contribution of this paper is as follows: (1) we rigorously test the performance of two baseline deep CNN architectures with five popular color normalization methods on three multicenter datasets with unique color characteristics. (2) We propose a novel metric that quantifies the color intra-variability tasets. (3) We propose an ensemble segmentation method that utilizes the un-normalized and CN-based models as weak learners contributing to a single segmentation prediction. (4) Analyze the effects and impact of color normalization on deep learning-based segmentation of nuclei in H&E.

The rest of this paper is organized as follows. Methods and Materials used in this paper are reviewed in section II. In section III, we outline the Experimental Results, and in section IV and V present Discussions and Conclusions.

## Materials and Methods

### Data

The first dataset, “TCGA-Kumar,” used in this paper was adapted from Kumar et al., and is publicly available (Kumar et al., 2017). The data is comprised of a diverse set of H&E stained tissue images with manually annotated nuclei, boundaries, and background labels. The annotations were created by undergraduate students then assessed by a pathologist for accuracy. The whole-slide images were digitized under 40X magnification and were obtained from *The Cancer Genomic Atlas* (TCGA). Twenty-nine regions of interest (ROIs) were cropped to a size of 1,000 × 1,000 pixels and were used in the development of CNN-models in this work. The following tissue types comprise the dataset: breast, lung, kidney prostate, bladder, colon, and stomach, and include both benign and diseased tissue samples. It presents a highly diverse dataset, with highly variable staining intensities, colors, and nuclei appearances across organs. Thus, this data is a good representation of the multicenter, multi-organ and multi-disease problem that could be common in clinical data. We chose to assess the CN and nuclei segmentation results on the data provided by Kumar et al. for these reasons.

The second dataset, “TNBC,” used in this work was first introduced in Naylor et al. (2017) and can be found at: https://github.com/PeterJackNaylor/DRFNS/tree/master/datafolder. The dataset is comprised of H&E stained, triple negative breast cancer (TNBC) ROIs, sampled from whole-slide images (WSIs) of 11 different patients. Approximately 3–7 images of size 512 × 512 were cropped from each WSI resulting in a total of 50 images. The dataset has a total of 2754 annotated cells where a sample ROI contains a minimum of 5 to a maximum of 293. To our knowledge, scanner information and acquisition parameters such as magnification were not available.

The third dataset, “SMH,” used in this work was sourced from St. Michael's hospital in Toronto, Ontario, Canada. This dataset is comprised of two sets: (i) 30 non-overlapping ROIs of size 1000 × 1000 cropped from the WSI of a single patient, and (ii) 12 512 × 512 non-overlapping ROIs cropped from the WSIs across two patients. The first dataset is used for color normalization using CycleGAN, “SMH-norm” and the latter is used for segmentation evaluation, “SMH-seg.” The WSIs are of sampled lymph nodes to search for metastases from a primary breast tumor. The slides were stained using H&E and digitized with an Aperio Scanscope AT Turbo whole-slide scanner under 20X magnification. The nuclei were annotated by an undergraduate assistant.

### Experimental Design

**Figure 1:**
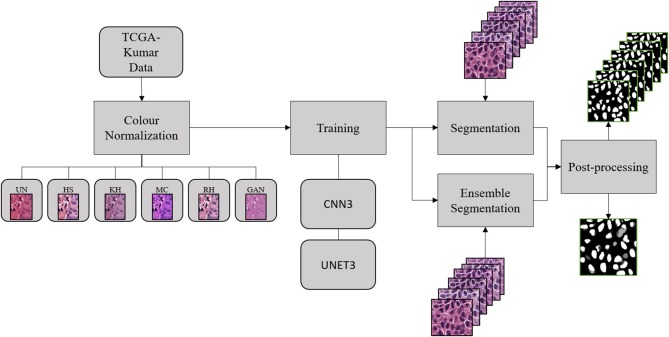
Experimental design.

Figure 1 depicts the experimental design used to address our research question. First, the TCGA-Kumar data was organized between un-normalized and normalized data. Subsequently, the datasets were used to develop the deep CNN models in the *training* stage. Using the developed models, *segmentation* was performed on the validation images followed by *post-processing*. This section will discuss each of these steps.

### Color Normalization

Due to the numerous CN methods used in computational pathology tasks (non-DL and DL-based segmentation frameworks), segmentation accuracy will be evaluated using several state-of-the-art CN methods. For each experiment, a different CN method was applied to the datasets before the CNN was trained. CN methods used in this paper were adapted from the *Stain Normalization Toolbox* made publicly available by the Department of Computer Science at the University of Warwick (Magee, 2014). Each method in the toolbox transforms the input image to a standard color space based on a reference image. The ability to maintain an accurate representation of stains in the CN output varies between each method. Early CN methods, such as histogram specification (“HS”) or color transfer (“RH”), have been noted to offer inaccurate representation of H&E concentrations (Magee et al., 2009). Other methods that perform stain deconvolution first, such as stain specific color transfer (“KH”), and spectral matching (“MC”) tend to offer a more accurate description of the individual stain components. This is largely due to the fact that stain deconvolution offers a robust and accurate description of constituent pure stains contained in the tissue specimen (Ruifrok and Johnston, 2001), which can be individually color normalized for more optimal results. Additionally, stain deconvolution removes color variability caused by unstandardized laboratory practices (Macenko et al., 2009). While, these methods have demonstrated their effectiveness in the past, because of their reliance on an expertly selected target image, other methods have been explored. Recently, general adversarial nets or GANs, have been adapted in digital pathology. Specifically, the CycleGAN or StainGAN, demonstrated superior results with respect to stain separation and maintaining image information compared to RH, MC, and KH after normalization was applied (Zhu et al., 2017; Shaban et al., 2019). Rather than relying on a single target image, the StainGAN maps a target image set to a reference image set. In this work, an open-source CycleGAN was used to perform the normalization operation (Erik Linder-Noren, 2018). The following subsections will further detail the methods used in this work.

#### Histogram Specification

*Histogram specification* or *histogram matching* includes the application of histogram equalization to the histogram of a query image and a reference image. Histogram equalization applies a transformation to an image such that the resulting image has intensity levels that are equally likely. Generally, equalization results in an image with increased contrast and increased dynamic range (Gonzales and Woods, 2008). In histogram specification, the transformation to a specified histogram is estimated. The transformation is then used to match the histogram of the individual red, green, and blue channels of the query image to the red, green, and blue (RGB) channels of the specified image. A recent work demonstrates that histogram specification can effectively transfer the color of a reference image to a query image and is validated quantitatively (Roy et al., 2019). However, due to multiple dyes and tissue structures that vary from image-to-image, histogram specification is known to introduce image artifacts such as incorrect stain mapping.

#### Color Transfer

During *color transfer*, after both the reference image and the query image are transformed to *lαβ* color space, the mean and variance of reference image are matched to that of the query image (Reinhard et al., 2001). Initially, this method was proposed for natural image color correction, but was later adopted in digital pathology for color normalization tasks. The stain normalization toolbox utilizes two variations of color transfer (1) the original Reinhard et al. method, and (2) a non-linear mapping approach that also employs image-specific color deconvolution (Khan et al., 2014).

##### Stain deconvolution

For CN tasks, stain or color deconvolution is used to transform an RGB-image to a stain space where the image channels are representative of the constituent pure stains contained in the tissue specimen (Ruifrok and Johnston, 2001). According to Ruifrok and Johnston (2001), separating the pure stains is possible through the Lambert-Beer law:

(1) $$\begin{array}{l} I_{p}=I_{O}e^{-\varepsilon_{p}c_{p}} \end{array}$$

where *I*_*p*_ is the source image, *I*_*O*_ is the background brightfield, *c* is the concentration of the dye pigment, *p*, and ε is the molar absorption coefficient (Haub and Meckel, 2015). The absorbance and mixing of stains can be modeled as:

(2) $$\begin{array}{l} OD=-\ln\left(\frac{I_{p}}{I_{O}}\right)=\underset{p}{\sum }\left(\varepsilon_{p}∙c_{p}\right)=VS \end{array}$$

(3) $$\begin{array}{l} S={OD^{*}V}^{-1} \end{array}$$

where *OD* is the optical density values for each channel in the RGB color space, *V* is the stain vector, and *S* is the concentration of each stain (Macenko et al., 2009). If the stain vector can be estimated, the pure stains described by *S* can be determined from the OD values of the input image (Ruifrok and Johnston, 2001). The challenge of stain deconvolution, however, is robustly estimating the stain vectors *V*, which should be done adaptively for each image.

##### Image-specific color deconvolution and non-linear color normalization

Khan et al. proposed a stain normalization algorithm that first estimates the stain matrix of both the reference image and query image using stain color descriptors followed by spline based mapping of reference image's stain channels to the query image's stain channels (Khan et al., 2014). A review by Li and Plataniotis (2015) stipulates that for this method color variation in the images are generalized and not addressed separately. Furthermore, statistics in the stain channels are modified which may result in inaccurate representation of stain and tissue features.

#### Spectral Matching

The spectral matching method utilized by the stain normalization toolbox is based on the work of Macenko et al. (2009), which estimates the stain vectors using singular value decomposition. Subsequently, the maximal range which comprises the stain content is estimated from the stain vectors and transformed back to the OD space. Various works state that while this method preserves histological information, normalization is not robust when images contain large stain variations (Bejnordi et al., 2016; Roy et al., 2019). Furthermore, because this method only addresses stain variation, other causes of color disagreements may not be addressed (Li and Plataniotis, 2015).

#### Generative Adversarial Networks: CycleGAN

CycleGAN performs unpaired image-to-image translation by using a model consisting of two generators, *G* and *F*, and discriminator pairs, *D*_*Y*_, *D*_*X*_. The *cycle consistent* methodology outlines that if *G* can map *X* to domain *Y*, *G*_*X*_ : *X* → *Y*, then *F* can map *Y* to domain *X*, *F*_*Y*_ : *Y* → *X*. The output of such mapping functions are ŷ = *G*(*x*) and $\hat{x}=F\left(y\right)$, respectively, where ŷ is a mapping of *X* in the *Y* domain, and $\hat{x}$ is mapping of *Y* in the *X* domain. The generators, *G*_*X*_, and *F*_*Y*_, are trained to generate images of the opposite domain, while the discriminators, *D*_*Y*_, *D*_*X*_ verify if the output images come from the real domain. This forward and backward cycle is achieved by the introduction of two cycle consistency losses represented as *L*_*Cycle*_. Combining these losses with adversarial losses on domain *X* and domain *Y* achieves unpaired image-to-image translation:

(4) $$\begin{array}{l} L=L_{Adv}+{\lambda^{*}L}_{Cycle} \end{array}$$

where, *L*_*Adv*_, is the adversarial loss and λ is the regularization parameter. Due to unpaired image-to-image translation, CycleGAN requires two data domains for training (Zhu et al., 2017; Shaban et al., 2019).

### Color Normalization Quality Metrics

Several metrics are used to determine the utility of color normalization. A common metric used to demonstrate color consistency, or lack thereof, is the normalized median intensity (NMI). However, NMI is more indicative of an image's intensity information rather than the color content. Therefore, in addition to NMI, we utilize other color metrics introduced in Roy et al. (2019) as well as normalized median hue (NMH), which is a novel proposed color consistency metric that measures variability in the hue across datasets.

The goal of these metrics is to demonstrate the variation of color across a population of images before and after CN was applied. By analyzing the variation of color after normalization, the ability of the applied method to transform an image set into a common space can be quantified. Several metrics are used, as described below and the coefficient of variation (CV) is quantified across the population of images for each metric. The CV is defined as the standard deviation divided by the mean of each metric from a dataset. For optimal CN results, the population variability would be low, with an optimal value for the metric (i.e., mean).

#### Normalized Median Intensity

The NMI of an image population quantifies the intensity variation of an image population and is used to compare the various CN methods (Bejnordi et al., 2016). NMI is defined as:

(5) $$\begin{array}{l} \text{NMI(I)}=\frac{Median_{i\in I}\left\{A\left(i\right)\right\}}{P_{95}\left\{A\left(i\right)\right\}} \end{array}$$

where the numerator is the median of the mean R, G, and B channels, *A(i)*, for the pixel, *i*, in image *I*, and P_95_ is the 95th percentile. A population of images are considered more consistent when the CV of the NMI computed over the population decreases (Basavanhally and Madabhushi, 2013; Bejnordi et al., 2016).

#### Normalized Median Hue (NMH)

The NMH metric was inspired by the NMI metric introduced by Bejnordi et al. (2016). Instead of looking at pure intensity, the NMH looks at consistency in the hue and is defined as:

(6) $$\begin{array}{l} \text{NMH(h)}=\frac{Median_{i\in I}\left\{H\left(h\right)\right\}}{P_{95}\left\{H\left(h\right)\right\}} \end{array}$$

where the numerator is the median of the hue-channel of a hue, saturation, and value (HSV) image at some pixel *h*, and the denominator is the 95th-percentile of the hue-channel at some pixel *h*. In a similar way as NMI, the CV of the NMH quantifies the color variation of a population of images but looking at the median hue value. Low NMH CV indicate less color intra-variability within an image population.

**Figure 2:**
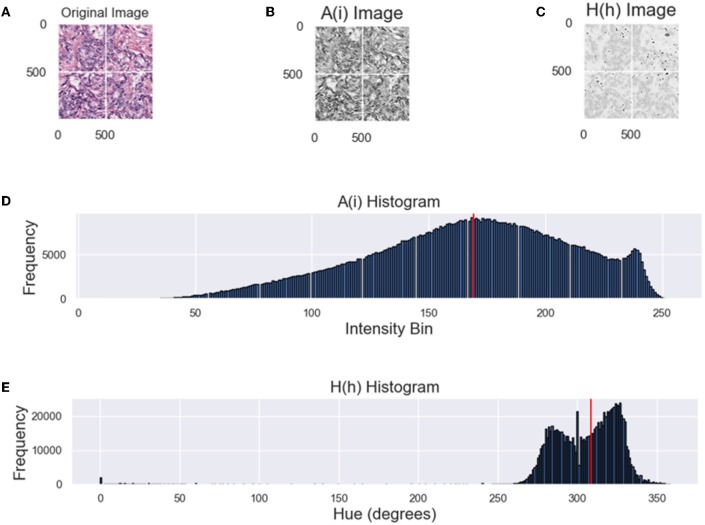
Comparing the NMI and NMH distributions. **(A)** Original image, **(B)** A(i)—grayscale image of **(A)**, **(C)** hue image (from HSV space), **(D)** grayscale intensity histogram, **(E)** hue histogram, where the red line indicates NMI or NMH respectively.

While both metrics attempt to analyze constancy of an image population, from the distribution of intensities in Figure 2 it is difficult to infer the dominant color from the intensity histogram (top). By converting the RGB H&E image to grayscale, color information is lost as color is correlated between the RGB channels. For an HSV image, the perceived color is in the hue-channel and is based on the angular properties of the HSV color space. From Figure 2, it is apparent that the hue histogram is bimodal indicating two dominant colors or stains. In the context of digital pathology, hues attributed to hematoxylin are closer to blue (240°) and hues attributed to eosin are closer to red (0°/360°). Mixing of stains or varying concentrations are between these values. Therefore, the median value of the hue distribution will quantify the relative hue of images and should be approximately consistent across datasets if the CN was applied successfully. From the intensity histogram, only the distribution of pixel intensities can be inferred. When assessing the quality of color normalization, it is important to be able to quantify the *color intra-variability* of an image population, which will be measured through the CV of the NMH.

#### Absolute Mean Color Error (AMCE)

The AMCE of the α and β channels of the *lαβ* color space represents the mean global color difference between the target image and the processed image for the respective channels. The α- channel corresponds to the red and green components of the image's color, while the β-channel corresponds to the yellow and blue components of the image's color. *AMCE*_α_ and *AMCE*_β_ equations are given below:

(7) $$\begin{array}{l} AMCE_{\alpha }=|\frac{1}{W}\overset{W}{\underset{i=1}{\sum }}\mu \left(\alpha_{i}\left(tar\right)\right)-\frac{1}{W}\overset{W}{\underset{i=1}{\sum }}\mu \left(\alpha_{i}\left(proc\right)\right)| \end{array}$$

(8) $$\begin{array}{l} AMCE_{\beta }=|\frac{1}{W}\overset{W}{\underset{i=1}{\sum }}\mu \left(\beta_{i}\left(tar\right)\right)-\frac{1}{W}\overset{W}{\underset{i=1}{\sum }}\mu \left(\beta_{i}\left(proc\right)\right)| \end{array}$$

where α_*i*_ (*tar*) is the *target* image information at some local ith window and α_*i*_ (*proc*) is the *processed* image information at some local ith window. The absolute difference is taken for these metrics and averaged by the total number of windows, *W*. A low AMCE value indicates similar color content between the target and processed images (Roy et al., 2019). Roy et al. stipulates that the global color of the reference image should be approximately equal to the global color of the processed image, which should be captured by the AMCE.

#### Contrast Difference (CD)

Contrast difference is a grayscale-base metric, where the change in contrast is quantified between the normalized image and the un-normalized image. In this work we adopt Roy et al.'s definition of CD:

(9) $$\begin{array}{l} CD\left(N,UN\right)=\frac{1}{W}\overset{W}{\underset{i=1}{\sum }}\frac{\sigma \left(N_{i}\right)}{\mu \left(N_{i}\right)}-\frac{1}{W}\overset{W}{\underset{i=1}{\sum }}\frac{\sigma \left(UN_{i}\right)}{\mu \left(UN_{i}\right)} \end{array}$$

where σ (*N*_*i*_) and σ (*UN*_*i*_) are the standard deviations of the normalized and un-normalized images at some *ith* window, and μ (*N*_*i*_) and μ (*UN*_*i*_) are the means. Regarding CD, Roy et al. hypothesizes that the contrast of the normalized image should be greater than that of the un-normalized image. Therefore, post-normalization a positive CD value would indicate an increase in contrast. Furthermore, Roy et al. notes that over contrast enhancement may result in discolouration of the nuclei and tissue structures. If the normalized image sets are used for clinical diagnosis, darker nuclei could be misinterpreted.

### Nuclei Segmentation Using CNNs

The CNN architectures utilized for nuclei segmentation were adapted from Kumar et al. (2017), “CNN3” and Ronneberger et al. (2015), “UNET3.” Unlike prior binary classifiers, which only discriminate nuclei against the background, these segmentation models were adapted to predict the nuclei and the corresponding boundaries at the same time. The method predicts the category of all the pixels of an image with only one pass. The input of the network is an H&E ROI and the output is the estimated classes. For CNN3 the output layer is a *softmax* function that is used during prediction to give the probability of the center pixel. The output of the model has three channels that represent the probabilities of each pixel being background, boundary or nuclei. For UNET3, the output layer is three-channeled and is achieved through convolution with sigmoid activation. Similar to CNN3, each output channel of UNET3 represents the probability of pixels being background, boundary or nuclei. Contrary to the method of Kumar et al. where a threshold applied to the *fuzzy* output to separate the classes, in our implementation of CNN3 and UNET3 the maximum probability is used to generate a binary map for each class. Nuclei class images are then refined via a simple and fast post-processing procedure.

#### Ensemble Segmentation

Ensemble segmentation has demonstrated benefit in digital pathology segmentation tasks (Naylor et al., 2017; de Bel et al., 2018). Traditionally, collections of networks are trained on the same dataset, and each model is then used to make a prediction. The predictions by each model are then averaged. The added benefit of using ensembles is to reduce the variance of the predictions, where each model can contribute to the prediction. In this work, the models trained on un-normalized and normalized data are used as an ensemble, where their predictions are averaged to produce a final segmentation. For instance, from Equation 10, let *n* represent models that were separately trained on the same dataset, but each dataset had a different color normalization method applied, *i* represent the input or query image for segmentation, *P*_*n*_(*i*) is the predicted class probability image of nuclei for image *i*, and *N* is the number of models.

(10) $$\begin{array}{l} E\left(i\right)=\frac{1}{N}\sum P_{1}\left(i\right)+P_{2}\left(i\right)..+P_{n}\left(i\right) \end{array}$$

**Figure 3:**
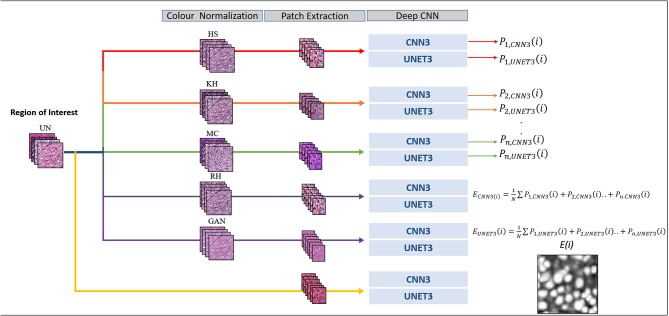
Segmentation framework.

By averaging the probability images of each model, the output probabilities represent the agreement or disagreement between models. Nuclei regions where probabilities overlap would be more prominent, while regions which have low agreement would be attenuated. To our knowledge, this is the first attempt from a color normalization perspective. The ensemble image is binarized using Otsu's threshold for the UNET3, and a fixed threshold for CNN3. The final prediction therefore reflects a contribution between all models. Normally a single CN method is applied to normalize data or is combined with the un-normalized training set. In these cases, any benefit of other CN methods, i.e., greater contrast, or improved color constancy, is ignored. Therefore, by including all un-normalized and CN models in the final prediction, improved performance is expected. Figure 3 below depicts the ensemble implementation.

#### Post-processing

The binary nuclei images predicted by the models are post-processed using a series of basic filtering and morphological operations. Initially, nuclei predicted images are filtered using a 3 × 3 median filter. Applying a median filter is a form of false positive reduction and attenuates single or very small pixel regions. This filtering operation is followed by morphological operations. Morphological operations in image processing apply a structuring element to an input image, creating an output image of the same size. For the proposed post-processing method, a structuring element is used to *fill* and *close* gaps in nuclei to ensure there are no holes. Subsequently, very small objects that were not attenuated by the median filter are removed in the boundary images.

## Experimental Results

In this section the experimental setup, implementation details, segmentation validation metrics and results will be detailed. First, color normalization using the toolbox will be outlined, followed by normalization using CycleGAN. Next, data preparation, parameters, and hyper-parameters concerning the CNN3 and UNET3 architectures are described. Lastly, color normalization results are presented followed by segmentation results.

The first experiment involved the normalization of the all the datasets; TCGA-Kumar, TNBC, and SMH. The data is CN by each of the five tested methods: histogram specification “HS,” color transfer “RH” (Reinhard et al., 2001), “KH” (Khan et al., 2014), spectral matching “MC” (Macenko et al., 2009), and cycle generative adversarial nets “GAN” (Shaban et al., 2019). To examine likeness and similarity to the reference image sets and intra-population color variation, each of the image quality metrics are measured for the respective dataset, and the CV for each metric is measured over the population of images.

The next experiment involved the development of the deep CNN models. As previously stated, the TCGA-Kumar set was used to develop the deep CNN architectures for each CN dataset. This experiment results in six models for each of the DL architectures (5 CN and 1 unnormalized). In this stage, the ensemble classifier is also assembled, which takes the result from each model and combines the outputs. Segmentation performance for each of the models is measured using segmentation overlap quantities for the TCGA test dataset initially.

To demonstrate the clinical utility and generalizability to multicenter data, the models trained on the TCGA Kumar dataset are applied to two other datasets (new data) not observed during training—the TNBC and SMH datasets. These images are all from different studies, centers, etc. and contain color variability that is representative of the multicenter challenge.

### Color Normalization Reference Images

**Figure 4:**
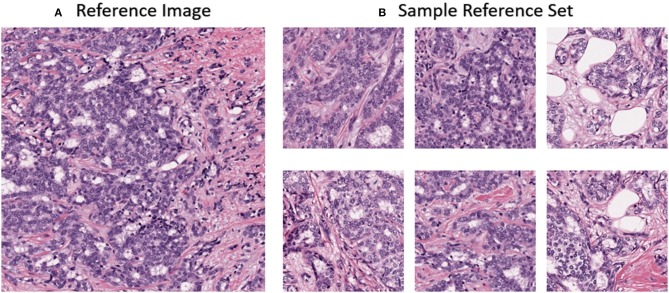
**(A)** Reference image for stain normalization toolbox **(B)** Sample dataset for CycleGAN training.

In this work, the color normalization toolbox (Magee, 2014) was used to apply traditional color normalization techniques to the datasets. Each method provided in the toolbox requires a reference image. The reference image was acquired from a ROI from St. Michael's hospital of a lymph node metastases secondary to breast invasive ductal carcinoma. The reference image was chosen due to its uniform color and high contrast and can be seen in Figure 4A. For normalization using CycleGANs, an image population of ROIs were used instead of a single reference image and can observed in Figure 4B.

### Cycle-GAN Implementation

**Figure 5:**
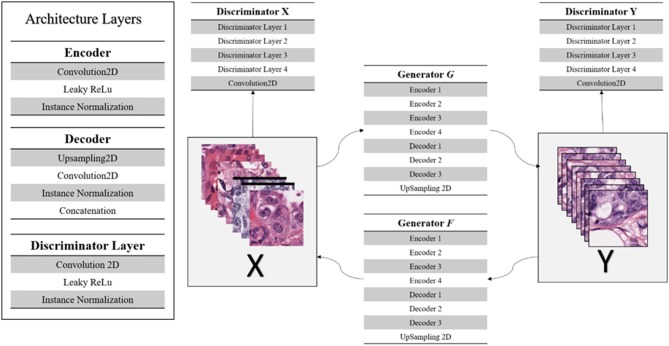
CycleGAN architecture.

The CycleGAN implementation used in this work uses a U-Net structured generator network of down-sampling and up-sampling paths, and a discriminator network of four discriminator layers comprised of a convolutional layer, Leaky ReLu activation and instance normalization (Erik Linder-Noren, 2018; Shaban et al., 2019). Figure 5 displays the CycleGAN architecture. In addition, most of the original architecture's hyper-parameters were maintained with λ = 10, learning rate of 0.0002, batch size of 1, and identity loss of 0.1^*^ λ (Zhu et al., 2017; Erik Linder-Noren, 2018). For our experiments, the un-normalized TCGA-Kumar, TNBC, and, SMH-Seg images are being translated to the SMH-norm domain. Note, while cases from the respective sets may be contained in both the SMH-seg and SMH-norm sets, non-overlapping patches were extracted and separated for the purpose of CycleGAN training and segmentation.

Due to the input size requirements of the CNN3 and UNET3 architectures, multiple CycleGAN models were trained. The CNN3 architecture requires a 1,000 × 1,000 × 3 input dimension and therefore needed to be trained on images of the dimension. Therefore, 30 1,000 × 1,000 × 3 ROIs of the SMH-norm data were used. Next, to train the CycleGAN to transform images from the TNBC data to the SMH-norm domain, 30 ROIs from the SMH-norm data were patched (non-overlapping) to match the dimensions of the TNBC data, 512 × 512 × 3. Therefore, 30 patches from the TNBC data and 30 patches from SMH-norm data were used to develop this model. Lastly, to transform the SMH-seg images to the SMH-norm domain, 12,512 × 512 × 3 images of the SMH-seg domain and 12,512 × 512 patches of the SMH-norm domain were used to develop the CycleGAN. Because the UNET3 architecture requires a 256 × 256 × 3 input dimension, the larger images for each dataset were simply patched to the appropriate size. Each model was trained for 100 epochs. The model which exhibited the best results qualitatively were chosen to represent the CN GAN images.

### Deep CNN Architecture and Segmentation

In this subsection, the experimental setup and implementation details for the nuclei segmentation models, as well as, the deep CNN architectures will be detailed. As a result of the unique input dimensions of the architecture's data preparation and training slightly varied and will therefore be outlined as well.

#### Data Preparation and Training Protocol for Nuclei Segmentation

Due to the numerous types of CNN architectures used in nuclei segmentation tasks, two architectures are used in our evaluation of CN on DL-based segmentation. The architectures to be evaluated are the standard CNN (Kumar et al., 2017), and the standard U-Net (Ronneberger et al., 2015). Observing the effect of CN on multiple architectures demonstrates the generalizability of our experiments. The data is CN by each of the five described methods (HS, RH, KH, MC, GAN). The un-normalized and CN datasets were then subsequently used to train each of the deep learning architectures (CNN3 and UNET). The optimized model for each dataset, along with the resultant ensemble model, are used to segment nuclei from test images.

While, both architectures aim to achieve the same goal, the data preparation and training process are slightly different. In both implementations the original images are “patched” into smaller images—for CNN3 51 × 51 pixels with a stride of 7, and for UNET3 256 × 256 pixels of non-overlapping patches. The CNN3 training protocol included a ~74% training and ~26% testing split, while the UNET3 implementation used a 59%% training, ~17% validation, and ~24% testing split. The testing data for both architectures included the same representative patches but differed by size and quantity to accommodate for the architecture design. In total, for CNN3 409146 51 × 51 patches were used for training and 147972 patches of the same size were reserved for the test set. In total, for the UNET3 architecture 292 256 × 256 patches were used for training, 60 patches for validation, and 112 for testing. Both data preparation protocols apply the same patch augmentations (90°, 180°, and 270° rotations), but mainly differ in the structure of their training labels. For CNN3, the center pixel of the 51 × 51 patch has a corresponding label as either background, boundary, or nuclei. However, for UNET3, each patch has a corresponding image label, where each pixel of the label is one-hot encoded for background, boundary, or nuclei. In addition, the original evaluation structure (Kumar et al., 2017) organized the test sets as *same organ* and *different organ* testing, where tissues under the latter category, stomach, bladder, and colon, were excluded from training. Therefore, to improve the generalization of our models at least one image of stomach, bladder, and colon were included in the training dataset. This training structure was maintained for UNET3 as well. After patching, the RGB images and the annotation data are applied to the CNN architectures to train the nucleus-boundary models.

Both architectures were trained on a personal computer (PC) equipped with a NVIDIA 1080 Ti graphics processing unit (GPU), 32 Gigibytes (GiB) RAM, 1 Terabyte (TB) hard-drive, and an Intel® Core™ i7-8700 CPU. The CNN model was implemented with Python using the PyTorch deep learning framework. The learning rate was set to 0.001, and unlike the original method in Kumar et al., the models were only trained for 40 epochs (~4-6 hrs) as accuracy did not increase significantly for greater epochs. All other parameters such as *batch size, drop-out*, and general architecture were maintained as in the original paper (Kumar et al., 2017). Unlike the CNN3 architecture, UNET3 was trained for a maximum of 100 epochs (~0.25 h). For the complete training algorithm refer to Figure 3 in section Ensemble Segmentation. Table 1 depicts, in high level, some parameters and hyper-parameters for the respective architectures.

**Table 1:** Description of deep CNN architectures.

**a) CNN3 architecture and description**				
**Layer name**	**Filter size**	**Activation**	**Dimension**	**Dropout**
Input image		–	51 × 51 × 3	–
Conv layer 1	4 × 4	ReLU	48 × 48 × 25	0.2
Max-pool layer	2 × 2	Max	24 × 24 × 25	–
Conv layer 2	5 × 5	ReLU	20 × 20 × 50	0.25
Max-pool layer	2 × 2	Max	10 × 10 × 50	–
Conv layer 3	6 × 6	ReLU	5 × 5 × 80	0.5
Max-pool layer	2 × 2	Max	3 × 3 × 80	–
Fully-connected	–	ReLU	1024 × 1	0.6
Fully-connected	–	ReLU	1024 × 1	0.6
Output layer		SoftMax	3	–
**Hyper-parameters**				
Optimizer: *stochastic gradient descent*				
Learning rate: 0.01				
Momentum: 0.9				
**b) UNET3 architecture and description**				
**Layer name**	**Input dimension**		**Output dimension**	
Input image	–		–	
Encoder network	256 × 256 × 3		8 × 8 × 512	
Center	8 × 8 × 512		1 × 1024	
Decoder network	1 × 1024		256 × 256 × 32	
Output layer	256 × 256 × 32		256 × 256 × 3	
**Hyper-parameters**				
Optimizer: *Adam*				
Learning rate: 0.001				

### Segmentation Validation Metrics

A series of overlap metrics are used to quantify segmentation performance. Firstly, the dice similarity coefficient (DSC) is used to evaluate the nuclei segmentation model's performance (Dice, 1945) since it accounts for the overlap between automated and manually segmented objects.

(11) $$\begin{array}{l} \text{DSC}=\frac{2TP}{2TP+FP+FN} \end{array}$$

A higher DSC indicates better segmentation accuracy compared to a lower value. The Extra fraction (EF) is another metric used to evaluate model performance. The EF quantifies over-segmentation and can be found by:

(12) $$\begin{array}{l} \text{EF}=\frac{FP}{TP+FN} \end{array}$$

Ideal model performance would demonstrate a high DSC value and a low EF value. Another metric commonly used to quantify segmentation performance is the Jaccard (JAC) Index or *intersection over union*, which measures the relative overlap between the segmented image and the corresponding label (Kumar et al., 2017). Given the nuclei image label, *L*, and nuclei prediction, *P*, the Jaccard Index is calculated as:

(13) $$\begin{array}{l} JAC\left(L,P\right)=\frac{\left|L\cap P\right|}{\left|L\cup P\right|} \end{array}$$

A greater JAC value would indicate greater similarity between the label and the prediction. Lastly, precision and recall additional metrics used to quantify segmentation performance and are defined as follows:

(14) $$\begin{array}{l} Precision=\frac{TP}{TP+FP} \end{array}$$

(15) $$\begin{array}{l} Recall=\frac{TP}{TP+FN} \end{array}$$

*Precision* quantifies the proportion of nuclei pixels in the prediction image that correspond to nuclei pixels in the label image, while recall is the proportion of nuclei pixels in the label image that were successfully detected by the prediction image. A low precision score indicates over-segmentation while low recall scores indicate under-segmentation.

### Color Normalization Results

**Figure 6:**
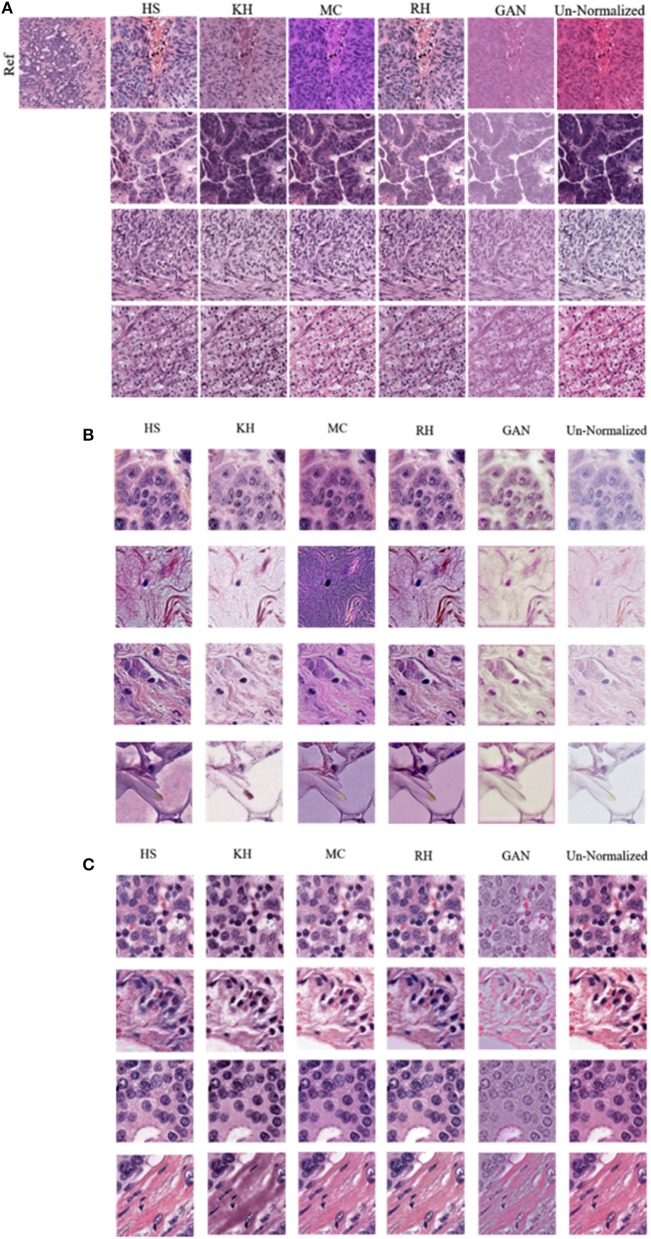
Comparison of various color normalization methods. **(A)** TCGA-Kumar dataset **(B)** TNBC dataset and **(C)** SMH-seg dataset. First column represents the reference image, while the following columns represent the color normalization methods, with the last column as the un-normalized query image.

In this section, results of the CN schemes are analyzed with respect to the TCGA-Kumar, TNBC, and SMH-norm datasets. Each of the ROIs from all datasets were CN using one of five methods. For ideal CN results, the normalized image set should demonstrate low color intra-variability and high likeness to the reference distribution. Figure 6 compares query images along with their normalized results with respect to the reference image. It is noted that the quality of CN differs between CN methods and even between the normalized results of a single method. It is possible that for each CN method artifacts were introduced. Artifacts from CN would manifest as a stained brightfied background, or incorrect color in nuclei or tissue. Since H&E have affinity to certain tissue structures, hematoxylin should be found predominantly in the nuclei, whereas eosin should be found in the stroma or other tissues. Image populations that maintain accurate stain representations while being normalized, are predicted to result in better performing segmentation models than images which are color normalized inaccurately. Inaccuracies caused by color artifacts and overlapping stain regions are expected to negatively impact nuclei segmentation downstream.

Firstly, staining of the brightfield background, tint, or discoloration of nuclei are present in many of the normalized images across all the datasets. For instance, HS, and RH, which simply apply the color statistics of the reference distribution to the target (Reinhard et al., 2001; Gonzales and Woods, 2008), contain gray-blue color in the normalized TCGA-Kumar images and staining in the brightfield background and lipid structures in the TNBC dataset. As another example, RH and GAN normalized images of both the TCGA-Kumar and TNBC datasets, while having a stained brightfield, result in normalized images that exhibit a *tint* which effects the contrast between nuclei and surrounding tissue structures. Furthermore, MC normalized images across the TCGA-Kumar and TNBC datasets exhibits inconsistent color mapping for the query images as indicated by vibrant stain colors and incorrect color mapping of tissues from the reference images to the query images. In addition, while KH normalized images demonstrate a lack of likeness to the reference image, the mapping of color in nuclei and stroma appear to be more accurate than the other methods. With respect to the SMH-norm data, with the exception of GAN, all other methods demonstrate great similarity to the un-normalized dataset. This is expected as the reference image is of the same tissue type and sourced from the same institution. While not perfect, all methods manage to achieve some similarity between the color normalized images and some likeness to the reference image. This similarity is be better analyzed through the quantitative results.

To assess the quality of CN quantitatively, the metrics that were introduced by Roy et al. (2019) are used. Roy et al. stipulated that the global color of the target image should be similar to that of the processed image. This idea will be referred to as *likeness* and will be quantified using AMCE α and β. In addition, Roy et al. hypothesizes that image contrast should increase post-normalization.This hypothesis will be analyzed using CD. Lastly, in combination with Roy et al.'s hypotheses, the quality of CN is evaluated by analyzing the variability amongst the normalized image population. This notion will be referred to as *color intra-variability* and will be quantified using the proposed metric, NMH. Analyzing the coefficient of variation for these metrics enable us to assess the stability of the CN methods across the images of each dataset. Metrics with greater CV values indicate that the CN method is not consistent. For instance, high CV values for *AMCE*_α, β_ indicate that the CN method had a large variation in global color error when compared to the reference image. Furthermore, for both NMI and NMH, high values of CV indicate large variations in intensity and color amongst the images of each CN dataset. Lastly, positive and high values of CV for CD indicate that the CN method was inconsistent in producing images with greater contrast, while negative and high CV values indicate that there was some contrast enhancement. Lower and positive values for CD are ideal.

**Figure 7:**
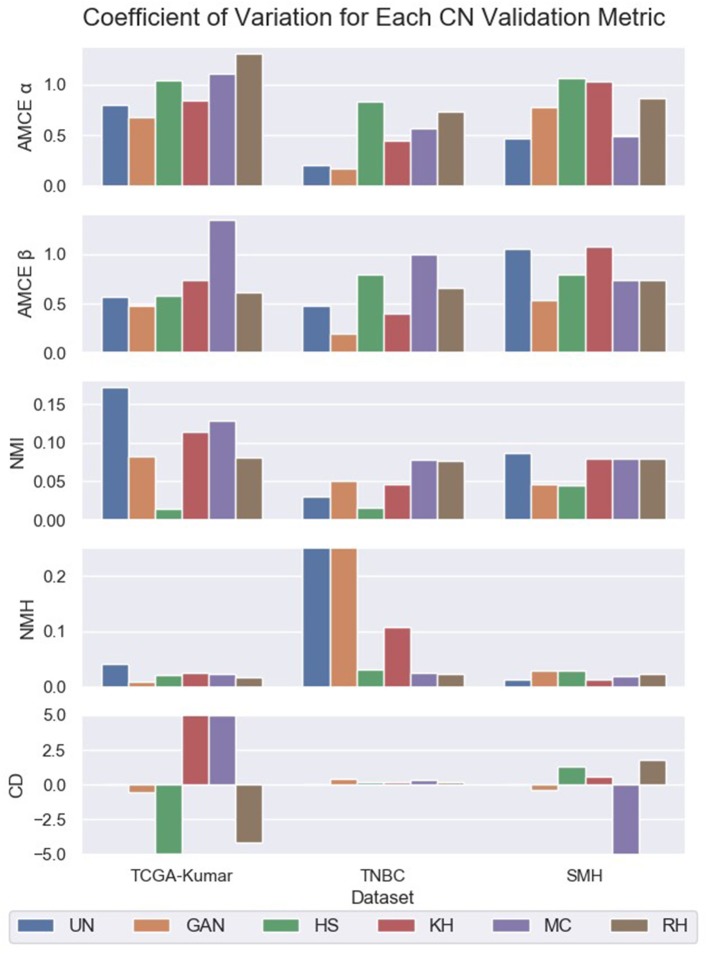
Coefficient of variation for unormalized and color normalized datasets.

The CV of NMI, AMCE, CD, and NMH were computed and compared amongst the CN image sets to the un-normalized set and is shown in Figure 7 and Table 2. Quantifying both the intra-variability of the image sets, and *likeness* to the reference distributions with these metrics by the CV will give a proxy measurement of the quality of CN. By knowing the quality of CN, we are hoping to infer which image sets may generate more robust segmentation models through indication of minimized variability within the dataset.

**Table 2:** Coefficient of variation for un-normalized and color normalized datasets.

	**AMCE α**	**AMCE β**	**NMH**	**NMI**	**CD**
**TCGA-Kumar**					
Method	5.788	4.330	0.139	0.593	2.863
GAN	**0.682**	**0.481**	**0.009**	0.082	**−0.568**
HS	1.039	0.580	0.022	**0.014**	–4.922
KH	0.847	0.742	0.025	0.115	7.543
MC	1.109	1.344	0.023	0.129	4.988
RH	1.311	0.618	0.017	0.081	–4.179
UN	0.800	0.565	0.042	0.172	N/A
**TNBC**					
GAN	0.172	**0.196**	0.824	0.051	0.407
HS	0.828	0.796	**0.031**	**0.016**	**0.146**
KH	0.447	0.396	0.108	0.047	0.216
MC	0.568	1.000	0.026	0.078	0.298
RH	0.732	0.661	0.023	0.077	0.147
UN	**0.205**	0.475	0.257	0.031	N/A
**SMH**					
GAN	0.775	**0.538**	0.030	0.046	–0.346
HS	1.068	0.797	0.030	**0.045**	1.332
KH	1.034	1.083	**0.014**	0.079	**0.544**
MC	**0.496**	0.739	0.019	0.079	–7.770
RH	0.870	0.734	0.023	0.080	1.766
UN	0.468	1.055	0.014	0.087	N/A

*Bolded values indicate methods which achieved the lowest value for each validation metric with respect to each dataset*.

Based on Figure 7 and Table 2 the HS, MC, and RH methods seem to produce less consistent CN results for TCGA-Kumar data, as demonstrated by the higher CV values for *AMCE* α and β, which indicates that global color characteristics are variable throughout the dataset. This is consistent with both the TNBC and SMH datasets except for KH, which is less consistent for the SMH dataset. With respect so NMI, most methods demonstrate intensity variability except for HS. In terms of color intra-variability (NMH), most methods demonstrate consistent color as defined by the median hue, except for the GAN method applied to the TNBC dataset. Lastly, CD difference appears to be highly variable for most methods of the TCGA-Kumar dataset and MC of the SMH dataset. The TNBC dataset shows stable CD post-normalization. With respect to segmentation performance, datasets which exhibit low variability in their CN metrics are predicted to outperform models whose datasets demonstrate large color variabilities.

### Nuclei Segmentation Model Performance

We tested two commonly used baseline architectures for evaluating the effect of CN on nuclei segmentation; the CNN3 and UNET as outlined in section Experimental Design, in addition to the results obtained by un-normalized images and the ensemble classifiers. The TCGA-Kumar data was used to develop the models. Training was implemented using 409146 representative patches from 22 ROIs, and 147972 representative patches from 7 ROIs of mixed tissue types was used for testing. To investigate the generalization of CNN models that use CN, 46 patches from 12 ROIs of triple negative breast cancer tissue, and 48 patches from 12 SMH lymph node metastases ROIs images were also tested. These datasets effectively demonstrate multi-institutional data with more than 21,000 nuclear boundaries annotated for TCGA-Kumar, 2754 cells for TNBC, and 1459 cells for SMH-seg. Furthermore, these datasets exhibit unique staining and color properties which make them ideal for our experiments.

**Figure 8:**
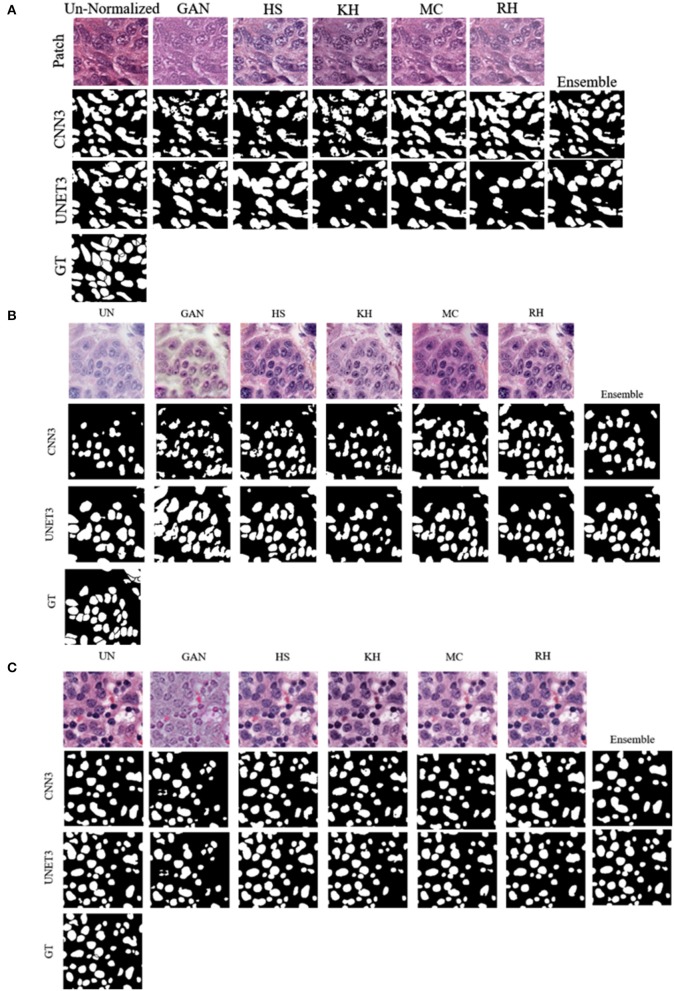
Sample segmentation result across all datasets **(A)** TCGA-Kumar **(B)** TNBC **(C)** SMH-seg.

**Figure 9:**
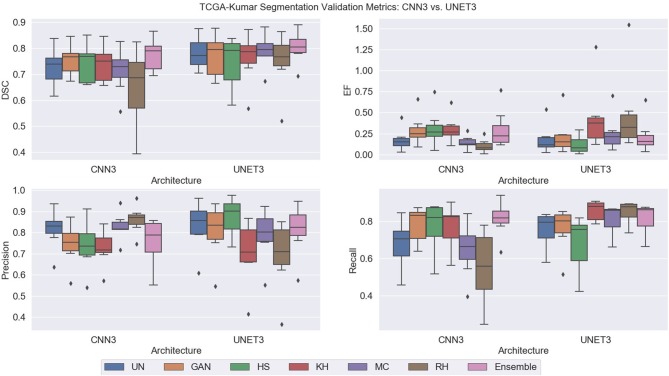
TCGA-Kumar segmentation validation metrics.

**Figure 10:**
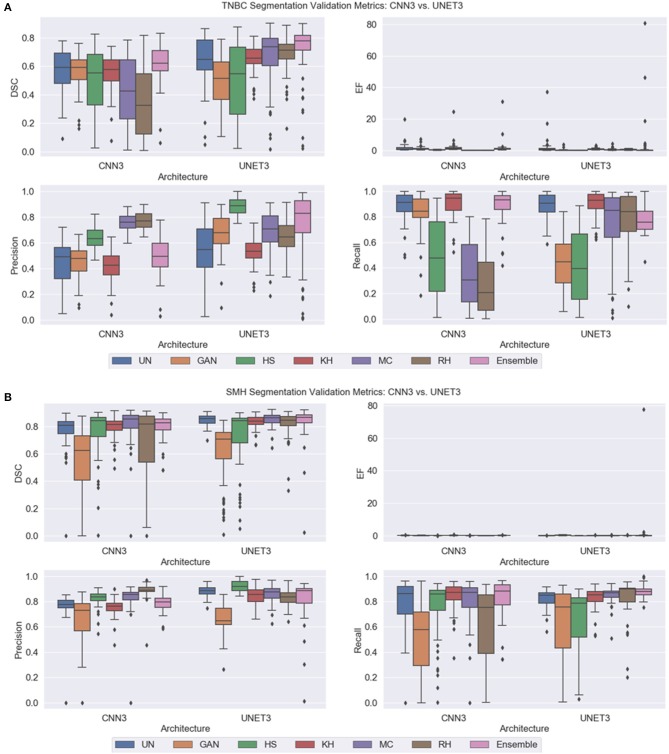
Segmentation validation metrics on new data. **(A)** TNBC dataset **(B)** SMH-seg dataset.

Note, for testing the UNET3 architecture on TGCA-Kumar data, representative non-overlapping patches were extracted, segmented, and then re-combined to measure segmentation performance. In addition, when testing the CNN3 on the TNBC and SMH-seg datasets, the same patches were extracted but zero-padded to match the input size of the CNN3. While training, a model was created at each epoch to select the most optimal epoch. To select the optimal models, segmentation performance as a function of epoch was plotted and analyzed. CNNs that were trained on un-normalized, GAN, HS KH, MC, and RH, models demonstrated the greater accuracy on the test set at epoch 16, while For UNET3, greater results were observed at epoch 40. Therefore, the models used at these epochs were used for segmentation evaluation. Figure 8 depicts a test image compared to the ground truths across all datasets. The first row depicting a sub-patch of an ROI while subsequent rows in Figure 8 depicts the predictions by the deep CNN architectures and the Ensemble. The binary masks from the output are the compared to the ground truths over all models and datasets using the average *Dice Similarity Coefficient (DSC), Jaccard Index (JAC), Extra Fraction (EF), Precision (PR), and Recall (RC)* over the respective dataset in Figures 9, 10.

#### Performance on TCGA-Kumar Dataset

Using DSC as the main performance metric, over both architectures, the Ensemble segmentation method produced the highest and most consistent DSC as depicted in Figure 9. From Figure 8A, ensemble segmentations are more refined compared to other methods and there is less noise in the segmentation. This is likely due to the fact that the result combines the most common attributes among the methods, which perhaps are the most reliable and consistent features across the datasets. In terms of the poorest performance in CNN3, the RH, and MC demonstrated the lowest mean DSC values and/or the highest spread indicating lower reproducibility. The HS method also had a high standard deviation on the CNN3 model. Similarly, in the UNET architecture, the RH method produced the lowest mean DSC, albeit with a relatively low standard deviation, compared to that of the HS method. Observing the segmentations qualitatively in Figure 8A, RH and HS have more difficulty with clustered or overlapping nuclei. Analyzing these results with respect to the CN metrics, HS, MC, and RH, exhibited the greatest variability in producing images that were similar to the reference image. This is apparent for the *AMCE* α and β values, which indicates there is a wide variability in the global color characteristics. This information is not available using the traditional NMI metrics, as shown, since the intensity variability is low on the HS method, but this method does not produce optimal segmentation results. This further demonstrates that intensity based metrics may not be sufficient in quantifying CN performance (and therefore, does not relate to downstream processing such as nuclei segmentation). Comparing these results to that of the un-normalized data; the CV for the ACME metric is higher for the un-normalized data compared to for example, the GAN method, and as seen by the segmentation results, the un-normalized data models are performing worse that than of the GAN models. This may be further indicated by the low color intra-variability as defined by the NMH. It is clear that the un-normalized data has the highest intra-variability with respect to the median hue value, and this variability may translate to poorer segmentation performance. That being said, despite the variability in the un-normalized data, there is still modest generalization of the CNN3 and UNET3 architectures on this dataset. In general, the CNN3 architecture had lower and more variable DSC values than the UNET3 architecture, indicating that the UNET provides more reliable and consistent segmentation results for this dataset. With respect to EF, the UNET3 seemed to have some issues with under segmentation (i.e., false positives) as KH and RH for this architecture have a larger and greater spread of values compared to other models. For CNN3, the Ensemble model had a greater spread of EF values whereas RH over-segmented images the least.

#### Performance on TNBC Dataset

Compared to the TCGA-Kumar set, both deep CNN architectures across all the models observed a lower performance on the TNBC testing set (Figure 10A), which suggests some generalization issues. Despite this, combining segmentations from all predictions in the Ensemble model achieved the highest and most consistent DSC compared to the other models, suggesting the combination of the results from the various models and datasets has the effect to increase signal, while suppressing noise. In the CNN3 model, the poorest performing models were from the RH, HS, and MC data, demonstrated by low mean DSC values with wide variance indicating sub-optimal segmentation and low reproducibility. These trends may be explained by the higher CVs in the ACME metrics, indicating that there is global color differences among the image populations that create generalization challenges for the CNN3 model. Similarly, in the UNET3 architecture, the GAN and the HS methods produced the lowest and widely varying DSC. This may be explained by considering the NMH and ACME metrics together, which show that there is a large variance among the images in terms of the global color characteristics and median hues. These results are consistent with what was previously observed for TCGA-Kumar segmentation. In terms of comparison with un-normalized data, it is interesting to note that the CV of the ACME and NMH metrics are lower than some of the other CN methods, and this un-normalized dataset achieved moderate performance for both CNN3 and UNET. This could mean a series of things. Firstly, the CN may be modifying the images in an unfavorable manner by increasing the global color variability in the image population, and the level of color variation in the original unnormalized data can be handled by these two architectures. In general, as found before, the UNET3 has higher performance over most methods and more consistently demonstrates higher generalization capabilities. Analyzing the EF results, each model seemed to have an issue with under-segmentation as the spread of EF values are quite large. By observing (Figure 8B) for this specific case, UNET3 had difficulty with clustered nuclei, whereas CNN3 was able to segment individual nuclei more effectively. However, nuclei predicted by CNN3 contain more holes, especially for GAN and HS. Poor segmentation accuracy for both architectures could be attributed to unfavorable color artifacts introduced post-normalization. From Figure 6B, MC HS, and RH, introduce incorrect color to the tissue structures and background. Analyzing the CN metrics, these models demonstrate high variability in the *AMCE*, low color intra-variability, and no improvement in contrast. The low color intra-variability for these methods may indicate that an image's color is too consistent such that color is transferred incorrectly to nuclei and stroma. Therefore, the global color observed, though similar to the reference, is an inaccurate representation because of CN.

#### Performance on SMH-Seg Dataset

Based on this dataset, yet again, the ensemble segmentation model is one of the top performers for both architectures. However, interestingly, in this dataset, the performance of the ensemble classifier is close to that of the un-normalized data with some outliers for the CNN3 architecture. Observing the ACME metrics, it can be seen that the CV for the *ACME* α quantity is the lowest for the original un-normalized data, which may be explaining the poorer performance in the CN schemes (difference in red and green colors with red as dominant in H&E). In addition, the NMH is low on the un-normalized dataset, indicating low intra-color variability and therefore, could be explaining the good segmentation performance. In terms of poor performance, the GAN and RH have the lowest mean DSC and highest variance, indicating generalization issues in CNN3. Similarly, in the UNET architecture, the HS and GAN methods are the poor performers. Observing the CN metrics, there are some trends that are supporting these segmentation results, although it may not be as clear as the previous examples. Because the reference image was taken from the same institution of the testing images, perhaps the color across the images would be similar—and therefore, the metrics would be slightly in favor of these images. Despite this, it is still evident that there is wide variability in CN methods and most of the models demonstrate variable color error but maintain low color intra-variability. Regarding EF, the models exhibited low EF values except for the Ensemble method, which has an outlier that skews overall performance. Comparing the results qualitatively, prediction images are very similar for the case depicted (Figure 8C). Due to the similarity of each prediction, the Ensemble prediction is similar to the other methods.

#### Performance on Multi-Center Dataset

**Figure 11:**
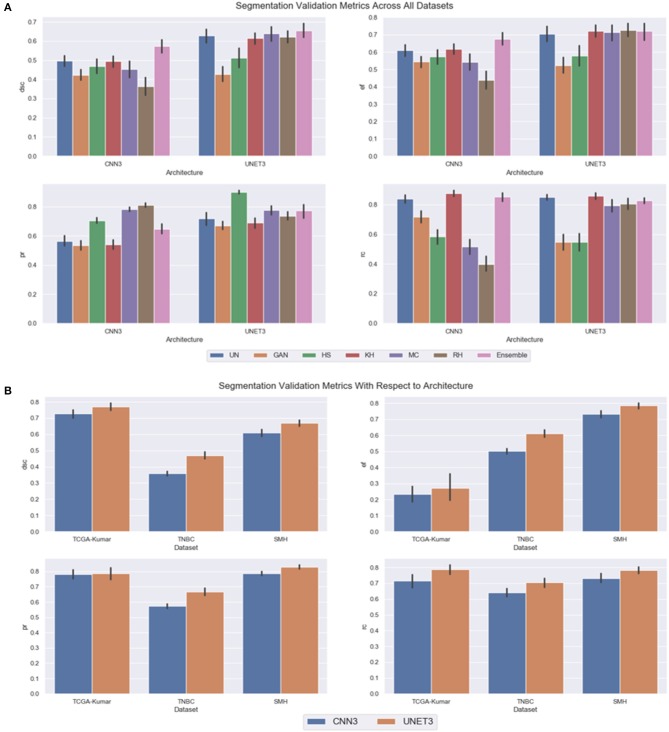
Segmentation validation metrics across all datasets, **(A)** CN model as a function of architecture **(B)** dataset as a function of CNN model.

In the previous subsections, segmentation performance was analyzed as a function of dataset and CN method. In this subsection, the results are analyzed overall to observe the optimal CN method per architecture, and overall generalization capabilities of each architecture. Firstly, instead of treating each dataset separately, the CN trends over all multicenter data combined are investigated by averaging the segmentation validation metrics over all three datasets. A greater perspective can be gained on which architecture across the different CN models performed better overall. Figure 11A depicts the segmentation performance across the multicenter data. Table 3 depicts the average segmentation validation metrics across the datasets. Bolded values indicate the top performing models.

**Table 3:** Mean validation metrics accross segmentation models and datasets.

**Validation metrics**	**Datasets**	**Architecture**	
		**CNN3**	**UNET3**
DSC	SMH-seg	0.609 ± 0.180	**0.669** **±** **0.132**
	TCGA-Kumar	0.727 ± 0.084	**0.771** **±** **0.084**
	TNBC	0.365 ± 0.159	**0.469** **±** **0.177**
EF	SMH-seg	**0.732** **±** **0.174**	0.784 ± 0.124
	TCGA-Kumar	**0.234** **±** **0.155**	0.271 ± 0.258
	TNBC	**0.508** **±** **0.184**	0.611 ± 0.188
Precision	SMH-seg	0.786 ± 0.100	**0.830** **±** **0.082**
	TCGA-Kumar	0.781 ± 0.092	**0.787** **±** **0.129**
	TNBC	0.566 ± 0.112	**0.667** **±** **0.161**
Recall	SMH-seg	0.732 ± 0.215	**0.783** **±** **0.143**
	TCGA-Kumar	0.715 ± 0.134	**0.788** **±** **0.094**
	TNBC	0.659 ± 0.188	**0.704** **±** **0.188**

*Bolded values indicate the architecture which achieved the best performance for each validation metric*.

Our results demonstrate that segmentation performance varied depending on the CN method applied. In CNN3, the MC, RH, GAN, and HS were the worst performing in terms of DSC over the multi-center datasets. The best performance was using the KH normalization, which was comparable to the un-normalized data. Inspection of the CN metrics in section Color Normalization Quality Metrics, show that low CV values for NMH or moderate CV values for ACME may describe this phenomena. An interesting point to mention regarding the CN metrics can be brought to attention here. Although the metrics vary greatly between datasets, we believe it is not possible to directly compare metrics across datasets, since the overall structure and color variability of the dataset will result in different “baseline” values for these metrics. Instead, it may be more pertinent to compare metrics within datasets that are generated by different normalization schemes. Segmentation performance was negatively impacted if CN reduced contrast or introduced color artifacts. Furthermore, false color, incorrect tissue stain localization, and clustered nuclei resulted in objects not being detected. This is especially evident if the original image has poor contrast, and the applied CN method does not improve contrast or increase object discernibility.

On average, the UNET3 architecture achieved a greater DSC than the CNN3 architectures indicating better generalization capabilities for UNET over all CN and un-normalized data models. In both architectures, the Ensemble method demonstrated the greatest average DSC, indicating that there is a synchronized averaging effect occurring—where the “signal” is being amplified, while the noise is being suppressed. Consistent with the previous results, under segmentation is most prominent for UNET (see EF), although it is only slightly higher than that of the CNN3.

Overall, it was found that segmentation models based on un-normalized datasets are comparable to models that use CN, especially in UNET. This demonstrates that even in the absence of a consistent color representation in the data, the deep learning models have still effectively learned how to discriminate nuclei and boundaries. This is an extremely interesting result—that despite wide color variability in the data, the models built from un-normalized data can still generalize. Therefore, we infer that the features the deep learning models learn may be color invariant, or that color features are weighted less when obtaining the pixel prediction. It also could mean that the features learned are relative colors, or abstractly related to color contrast, which does not take into account absolute colors. This phenomenon could be the result of using 2D convolutional filters on each of the R, G and B channels separately in the first layer. Color images are highly correlated, and color edges and textures are distributed across the RGB channels. Therefore, by using 2D filters, the inherent correlation across channels may be lost, which ultimately may cause the CNN to be less sensitive to color information. Therefore, if CN is applied for pre-processing, it is imperative to consider the risks of using CN instead of un-normalized data.

In Figure 11B, the graph is included as an average over all CN and un-normalized data models, to show overall generalization capabilities of both architectures as a function of dataset. Essentially, such analysis will differentiate which architecture is able to generalize to the validation sets. It was expected that the architectures would perform well on the TCGA-Kumar test set, as at least one ROI of each tissue type was included in the training data. Different from this, the TNBC data was of a tissue type observed during training but exhibited very dissimilar stain and color properties. In addition, the SMH-seg dataset was an entirely new tissue type not included in the training set, but showed some similarity to the stain properties of the training data. Furthermore, the training data was normalized using images from the SMH dataset so it is inferred that the architectures would generalize well.

As shown, and supported by other results, the UNET architecture performs the best overall datasets (with highest DSC) at a cost of a slightly higher false positive rate over the CNN3. It is shown that across datasets, the TCGA-Kumar is the best performing, which aligns with what was previously stated. The poorest performance comes from the TNBC data, and we believe this is largely because the original color characteristics were so different from the original training data. Observing Figure 6, the un-normalized TNBC images appear to be quite faint with low stain concentration in general across the example images. Compared to the un-normalized images of the TCGA-Kumar dataset, the TCGA-Kumar images appear to be highly concentrated with H&E staining. Furthermore, even compared to the SMH set, the SMH images appear to have high concentrations of H&E staining. Perhaps low CN performance for the TNBC set can be attributed to the stark differences in stain concentrations between the TNBC images and the image used to normalize them. As previously described, high CV for AMCE α, β values may negatively impact generalization. From this, we can conclude that the color or stain mapping from the reference image to the target set is not consistent across all datasets. This indicates that features or colors learned by the CNN models may be different from set to set, and despite the fact that normalization is applied, features can be altered thus impacting model generalization. These observations are consistent with the segmentation metrics in Figure 11B.

## Discussions and Conclusions

In this work, we evaluated the impact of color normalization on the complex task of segmenting nuclei for computational pathology applications. We applied common color normalization techniques to datasets that contain highly variable and unique staining properties, and evaluated the impact of normalization on popular baseline CNN architectures. At the same time, we utilized recent normalization metrics and proposed a novel one, NMH, which measures the color intra-variability of an image population. In addition, we proposed an ensemble segmentation method which uses individual CN models as weak learners to make joint predictions.

Our results demonstrate that coupling the NMH with the metrics proposed by Roy et al. (2019) can reveal interesting patterns which reflect the impact of color normalization on segmentation using CNN architectures. Observing variable AMCE and NMH values could be an indication if a CN method would result in desirable segmentation results. Only analyzing the NMI, which is traditionally used, does not indicate the color variability of an image population and may not be a desirable metric for quantifying the quality of color normalization.

However, despite the observed variability in the color normalization validation metrics, CNN models, especially UNET, are able to effectively segment nuclei especially when an ensemble prediction is used. The ensemble method outperformed all other models on average across all the datasets. In addition, our results reveal, that color normalization impacts CNN generalization, possibly as a result of features modified by the CN methods. For future works, more data for CycleGAN normalization, and additional architectures are desirable. By expanding this experimentation and investigating the many different CNN architectures, a better understanding of CNN features can be realized and tasks such as segmentation could be better understood.

## Data Availability Statement

The first dataset analyzed for this study can be found in Nuclei Segmentation Benchmark via https://nucleisegmentationbenchmark.weebly.com/dataset.html. The second dataset can be found via https://github.com/PeterJackNaylor/DRFNS/tree/master/datafolder. Digital lymph node specimens are not publicly available because: approval for the dataset to be made public has not been obtained from St. Michael's Hospital.

## Ethics Statement

Algorithms used in this paper rely exclusively on open source data, and a second repository that depends on the use of anonymized digital lymph node specimens that does not contain identifiable information. Therefore, Research Ethical Board (REB) approval for secondary analysis was not required (Tri-Council Policy Statement Article 2.4). However, approval for the dataset to be made public has not been obtained from St. Michael's Hospital.

## Author Contributions

JP implemented and evaluated the color deconvolution and normalization pipeline. JP and TG-T implemented and evaluated the deep learning method. Overall contribution to write and edit the manuscript is as follows, JP 40%, AK 30%, KJ 8%, DA 8%, TG-T 8%, and ED 6%.

### Conflict of Interest

TG-T is employed by company, Pathcore Inc. The remaining authors declare that the research was conducted in the absence of any commercial or financial relationships that could be construed as a potential conflict of interest.
